# Development of an efficient veterinary rabies vaccine production process in the avian suspension cell line AGE1.CR.pIX

**DOI:** 10.1186/s12896-022-00747-5

**Published:** 2022-06-17

**Authors:** Khaled Trabelsi, Meriem Ben Zakour, Ingo Jordan, Volker Sandig, Samia Rourou, Hela Kallel

**Affiliations:** 1 Biotechnology Development group, Institut Pasteur de Tunis. Université Tunis El Manar., 13, place Pasteur. BP 74., 1002 Tunis, Tunisia; 2 ProBioGen AG, 13086 Berlin, Germany; 3 Department of Life Sciences, Health Biotechnology Program - King Fahad Chair for Health Biotechnology, College of Graduate Studies, Arabian Gulf University, PO Box 26671, Manama, Kingdom of Bahrain; 4 Laboratoire Teriak, Zone Industrielle, El Fejja Mornaguia, 1153 La Manouba, Tunisia; 5 Quantoom Biosciences, Nivelles, Belgium

**Keywords:** Avian cell lines, Rabies virus, Process optimization, Veterinary rabies vaccine

## Abstract

**Background:**

Mass vaccination of dogs as important rabies reservoir is proposed to most effectively reduce and eliminate rabies also in humans. However, a minimum coverage of 70% needs to be achieved for control of the disease in zoonotic regions. In numerous developing countries, dog vaccination rate is still dangerously low because of economic constraints and due to a high turnover in dog populations. Improved vaccine production processes may help to alleviate cost and supply limitations. In this work, we studied and optimized the replication and vaccine potency of PV rabies virus strain in the muscovy-duck derived AGE1.CR and AGE1.CR.pIX suspension cell lines.

**Results:**

The BHK-21-adapted PV rabies virus strain replicated efficiently in the avian cell lines without requirement for prior passaging. CR.pIX was previously shown to augment heat shock responses and supported slightly higher infectious titers compared to the parental CR cell line. Both cell lines allowed replication of rabies virus also in absence of recombinant IGF, the only complex component of the chemically defined medium that was developed for the two cell lines. After scale-up from optimization experiments in shake flask to production in 7-l bioreactors peak virus titers of 2.4 × 10^8^ FFU/ml were obtained. The potency of inactivated rabies virus harvest according to the NIH test was 3.5 IU/ml. Perfusion with the chemically defined medium during the virus replication phase improved the potency of the vaccine twofold, and increased the number of doses 9.6 fold.

**Conclusion:**

This study demonstrates that a rabies vaccine for animal vaccination can be produced efficiently in the AGE1.CR.pIX suspension cell line in a scalable process in chemically defined medium.

## Introduction

Rabies is an usually fatal viral zoonosis caused by negative-stranded RNA viruses of the *Lyssavirus* genus. While all mammals appear to be susceptible to the disease, carnivora and chiroptera are considered to be the most important reservoirs. Human rabies mortality exceeds 60,000 deaths every year worldwide and 99% of the cases occur in Africa and Asia [1]. The vast majority of human cases (> 90%) results from the bites of rabid domestic or feral dogs [2, 3] making the fight against canine rabies essential in the prevention of human rabies.

Parenteral killed vaccines and baited modified-live vaccines are available for control of rabies in animals [4]. The World Health Organization (WHO) considers mass dog rabies vaccination as the most effective method to prevent the transmission of the disease to humans [1, 5]. Canine rabies affects also cattle with estimates as high as 11,500 animals being killed by rabies per year in Africa alone [3]. At the estimated incidence of 5 deaths/100,000 cattle the agricultural impact amounts to $12.3 million annually in Africa and Asia [6]. Furthermore, studies have estimated the global burden of canine rabies to approximately 124 billion dollars per year [7].

Inactivated rabies vaccines have been shown to be safe, efficient and cost-effective means to control rabies in dog populations. Vaccination is also preferred to the extermination of dogs because mass culling has only short-term effects on the population size, may drive dogs into hidden or nocturnal habitats, open niches for other susceptible carnivora such as jackals or racoons, and may even replace a partially vaccinated with a younger, non-vaccinated population. Such inadvertent changes to the ecology of reservoir and vector may thus further complicate control of the virus [8, 9].

However, the vaccination rate of dogs in many developing countries remains far below the threshold of 70%, which is the WHO recommended vaccination coverage rate to break the cycle of virus transmission to humans [3, 10]. Limited resources and economic constraints in developing countries are among the main hurdles that impede canine rabies control [11]. Moreover, the high turnover in dog populations requires continuous efforts towards maintaining uninterrupted vaccination rates [12].

Injectable vaccines are produced in adherent cell lines such BHK-21 and NIL-2 [13, 14]. The conventional production processes depend on culture media containing animal serum and suitable surfaces for cell cultivation and processing. The complexity of such processes increases costs of goods and leads to a more expensive final product.

AGE1.CR (in the following CR) is a continuous cell line that was derived from primary cells of a muscovy duck embryo. Immortalization is maintained by stable expression of the E1A and E1B genes from the human adenovirus serotype 5 [15]. The CR cell line was further modified by introduction of an expression cassette for the structural gene pIX from human adenovirus to improve replication of certain viruses [15]. The CR and AGE1.CR.pIX (in the following CR.pIX) cell lines were adapted to proliferation in suspension in a medium free of animal derived components [16].

CR and CR.pIX cells were evaluated for the production of different vaccine viruses including influenza viruses [17], MVA (modified vaccinia Ankara virus) [18, 19], and different avian viral vaccine strains [20]. Compared to a conventional production process, the costs of goods calculation, efficacy and safety were recently reported to be highly favourable also for the production of a complex human vaccine based on CR.pIX suspension cultures in the corresponding chemically defined media [21, 22].

In this work, we studied the replication of the PV rabies virus strain in CR and CR.pIX cells. We expect that the use of cell lines growing in suspension and in a chemically defined medium will contribute to lower the overall cost of a rabies vaccine and to increase access to the vaccine in developing countries. For this purpose, we investigated the impact of the operating parameters on virus titer and estimated the potency of the experimental vaccine according to the NIH test.

## Results

### Permissivity of CR and CR.pIX cells to rabies virus

**Fig. 1:**
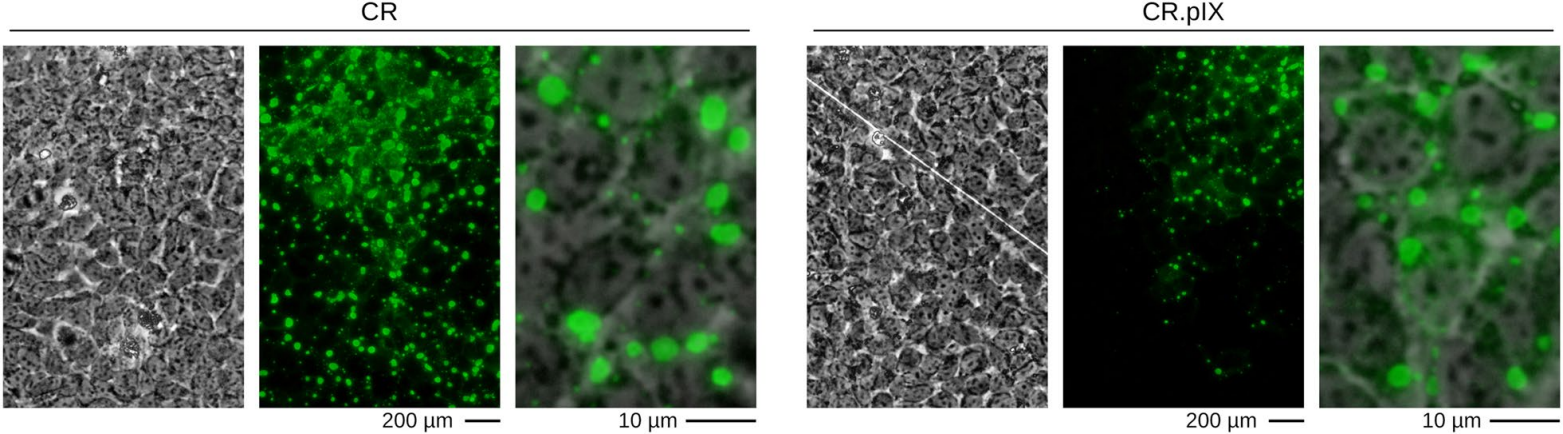
Immunofluorescence staining of CR (**A**) and CR.pIX (**B**) cells infected with PV rabies virus strain, 3 days post infection

The permissivity of CR and CR.pIX cells for the PV rabies virus strain were found to be similar in pilot experiments with adherent and suspension cultures in non-agitated flasks (data not shown). Immunofluorescence staining of adherent cells with anti-rabies nucleocapsid conjugate resulted in mainly cytoplasmic granules or inclusion bodies without prominent cytopathic effect (Fig. 1). The initial experiments furthermore suggested no losses or increases of virus titers after three passages of the virus (data not shown). PV rabies virus strain previously produced in BHK-21 cells was therefore used for all subsequent cell infections without adaptation of the virus.

After the positive initial results, a more stringent optimization was performed. CR and CR.pIX cells were grown in agitated shake flasks in CD-U5 medium and infected at an initial cell density of 2 × 10^6^ cells/ml, at 34 °C and an MOI of 0.01. CR° and CR.pIX° cells were infected under the same conditions, except that CD-U5 medium was not supplemented with the LONG R3IGF.

**Fig. 2:**
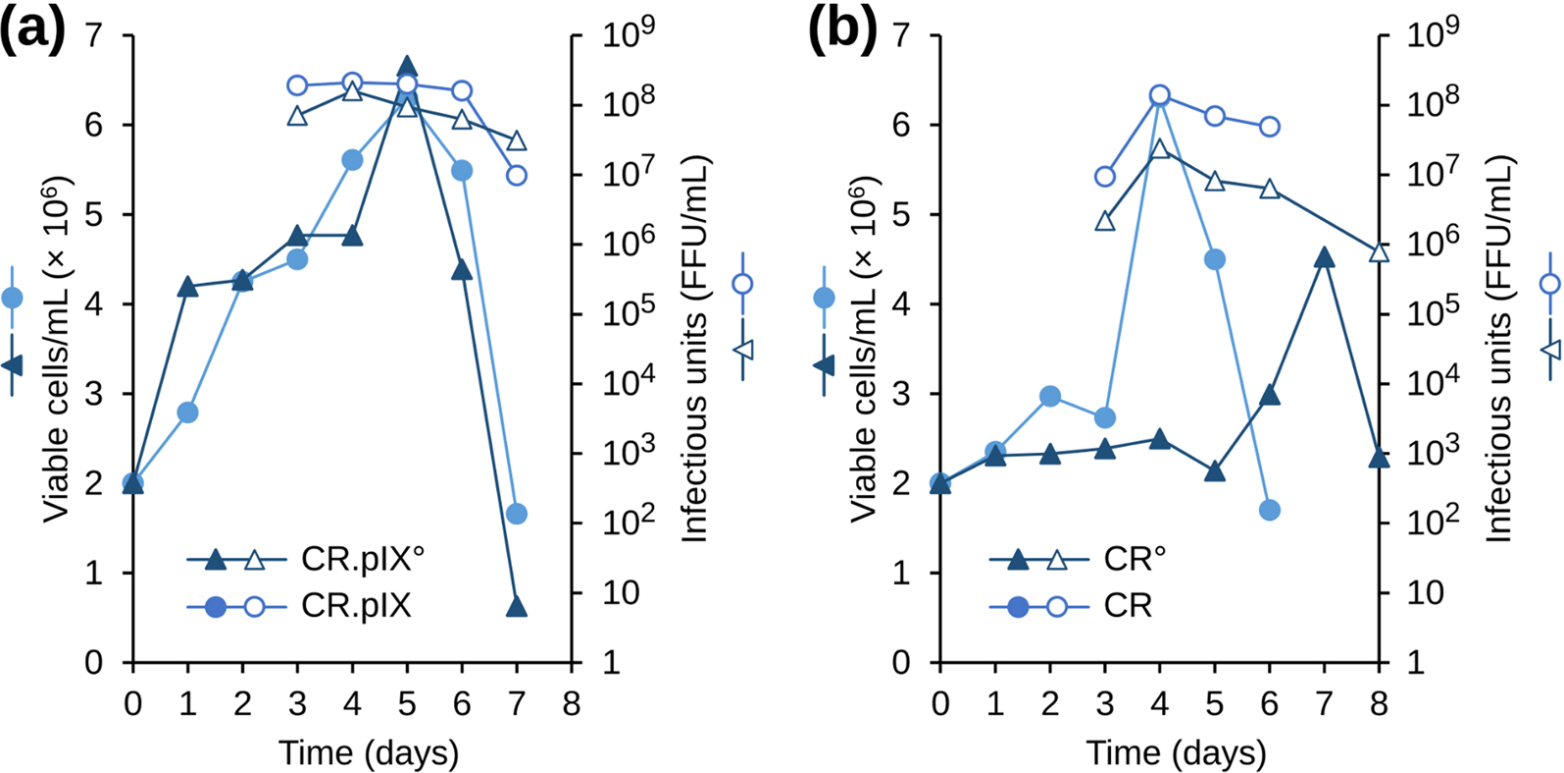
Kinetics of PV rabies virus strain replication in (**A**) CR.pIX and CR.pIX cells, and (**B**) CR and CR cells. Cells were infected at an MOI of 0.01 in CD-U5 medium

Kinetics of cell density and rabies virus titer of the various cultures are displayed in Fig. 2. Cell density profiles of CR.pIX and CR.pIX° cells were similar. Cells continued to proliferate 2 days into the infection but viability sharply decreases at day 5 as virus titers increase in parallel (Fig. 2A). At day 5, viable cell density level was around 6.5 × 10^6^ cells/ml for both cell lines. For CR.pIX° cells, the highest virus titer reached 1.6 × 10^8^ FFU/ml at day 4, then it slowly declined. At the end of the culture (at day 7), virus titer was equal to 3 × 10^7^ FFU/ml (Fig. 2). Kinetics of rabies virus replication in CR.pIX cells showed a similar profile that deviated only after peak titers were obtained. From day 3 to day 6, the virus titer reached a peak level of approximately 2 × 10^8^ FFU/ml and decreased 20-fold thereafter, a drop that was more pronounced for CR.pIX cells than CR.pIX°.

The viable cell profiles of CR and CR° cells after infection were not as favorable as those observed for CR.pIX cell lines (Fig. 2B). CR cell density increased to 6 × 10^6^ cells until day 4 but then decreased severely, whereas CR° cell density increased only gradually until day 7. Replication of PV rabies virus strain in CR cells resulted in a maximal virus titer of 1.4 × 10^8^ FFU/ml at day 4. The highest virus titer for CR° was 2.4 × 10^7^ FFU/ml, also at day 4 post infection, but tenfold lower compared to CR.

Compared to the parental CR cell lines, these data suggest that the CR.pIX and CR.pIX° cell lines allowed replication to higher infectious titers and exhibited more robust culture profiles after infection. CR.pIX and CR.pIX° cells may be more suitable for rabies virus production and were therefore used for subsequent studies.

### Effect of MOI

**Fig. 3:**
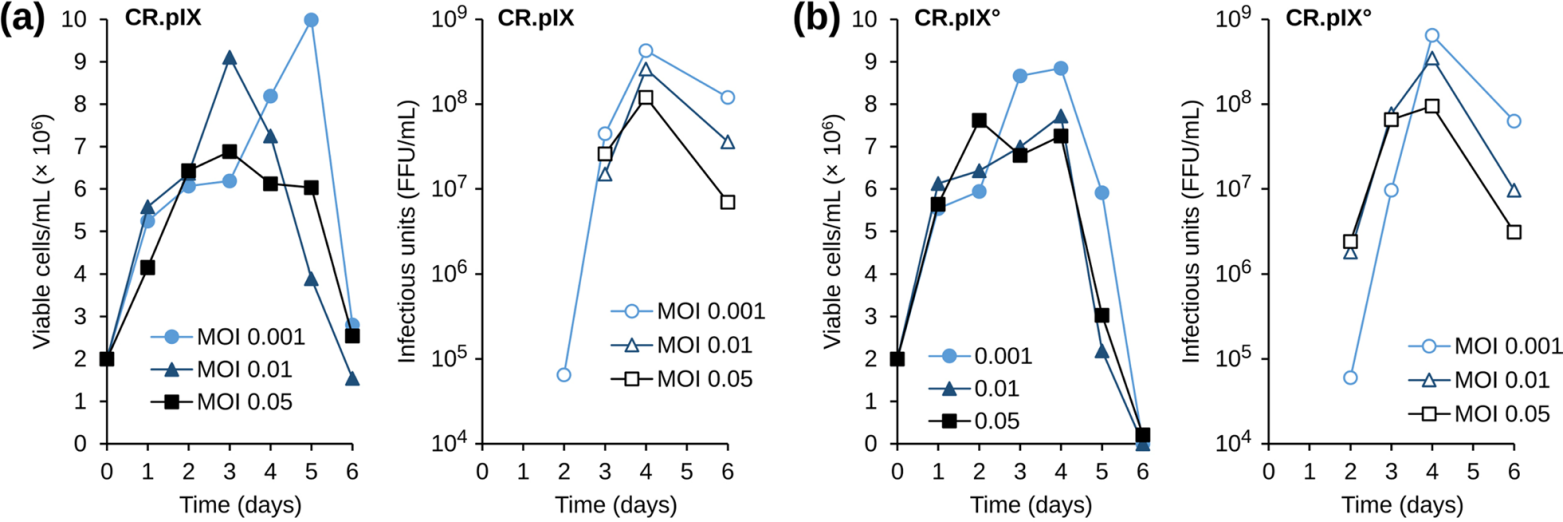
Effect of MOI on rabies virus replication in (**A**) CR.pIX and (**B**) CR.pIX cells

The two CR.pIX cell cultures were adjusted to 2 × 10^6^ cells/ml in the appropriate medium and infected with MOIs 0.001, 0.01 and 0.05 (Fig. 3). As observed in the previous experiment, cultures proliferated during the virus production phase at 34 °C until day 4 and viable cell density declined thereafter. The peak cell density correlated inversely with the MOI. The viral replication kinetic was similar under all tested conditions. Titers increased progressively, reached a maximum at day 4 post infection, and decreased thereafter. The maximum virus titer obtained in CR.pIX° was 6.8 × 10^8^ FFU/ml after infection with an MOI of 0.001. The yield in CR.pIX cells infected at the same MOI was 4.3 × 10^8^ FFU/ml. The drop in virus titers at the end of the culture interval appeared to be more pronounced for CR.pIX° (tenfold) compared to CR.pIX (fourfold). Cells infected at the higher MOIs showed lower virus titers (Fig. 3). In conclusion, an MOI of 0.001 appears to be optimal for production of PV rabies virus in both CR.pIX culture formats.

### Effect of cell density at infection

**Fig. 4:**
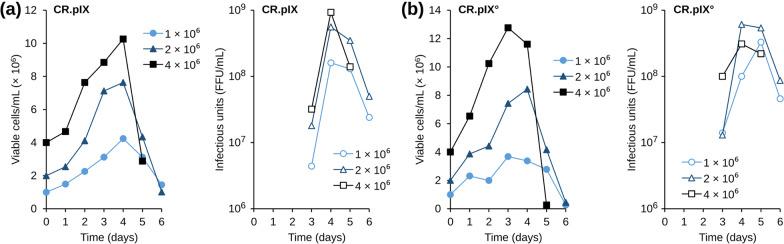
Effect of cell density level at infection on (**A**) CR.pIX° and (**B**) CR.pIX cells. Cells were infected at an MOI of 0.001 in CD-U5 medium without medium exchange

Cell density at infection can affect the kinetics of virus replication and accumulation in the culture medium. To test such effects, CR.pIX and CR.pIX° cells were infected with an MOI of 0.001 at the different cell densities 1 × 10^6^, 2 × 10^6^ and 4 × 10^6^ cells/ml. As summarized in Fig. 4, cells again continued to proliferate for up to 4 days after infection. Infection of CR.pIX° cells at 2 × 10^6^ cells/ml resulted in the highest virus titer of approximately 6 × 10^8^ FFU/ml. Virus titers reached 3 × 10^8^ FFU/ml when cells were infected at 1 × 10^6^ cells/ml or 4 × 10^6^ cells/ml. Although the virus titer peak was delayed to day 5 when the cells were infected at the lowest cell density, the specific virus productivity at 1 × 10^6^ compared to 4 × 10^6^ cells/ ml was higher (66 FFU/cell/day versus 19 FFU/cell/day). The highest productivity (76 FFU/cell/day) was achieved when the cells were infected at 2 × 10^6^ cells/ml.

CR.pIX cell infection at a high cell density level (4 × 10^6^ cells/ml) resulted in a virus titer of 9 × 10^8^ FFU/ml. Virus titers reached a maximum of 5.6 × 10^8^ FFU/ml and 1.6 × 10^8^ FFU/ml at day 4 when the cells were infected at 2 × 10^6^ cells/ml and 10^6^ cells/ml, respectively. The highest specific virus productivity (70 FFU/cell/day) was seen at 2 × 10^6^ cells/ml, similar to CR.pIX° cells.

This experiment suggests that IGF supplementation may augment rabies virus titers at high cell densities. Our data furthermore demonstrate that CR.pIX infection at the highest cell density enhanced rabies virus titer 1.6-fold although a slight drop of the specific virus productivity was observed (58 FFU/cell/day versus 70 FFU/cell/day).

### Effect of medium dilution

Medium design can be a critical parameter for optimal replication of a virus in a given cell line. One important medium property that can impact vaccine production processes is omsolalrty [23]. CD-U5 is a chemically defined medium optimized for maintenance of the avian cell lines in single cell suspension cultures. This medium is characterized by a low osmolality of 270 mOsm/kg. Basal media such as DMEM are optimized for adherent cultivation in presence of animal sera and are typically in the range of 320—360 mOsm/kg. To test whether addition of a medium with a higher osmolality influences yields of infectious titers, increasing amounts of DMEM were added to the infected cell culture.

CR.pIX and CR.pIX° cells were infected at a cell density of 2 × 10^6^ cells/ml, and an MOI of 0.001 at 34 °C. CD-U5 medium was diluted with DMEM at 25%, 50% and 75%. Undiluted CD-U5 medium was used as reference. Specific virus productivity was calculated when the peak of rabies virus titer was obtained at day 4 or 5 post infection and normalized to the specific virus productivity reached in CD-U5 medium. In addition, cell aspect and aggregation was monitored by daily observation by light microscopy.

**Fig. 5:**
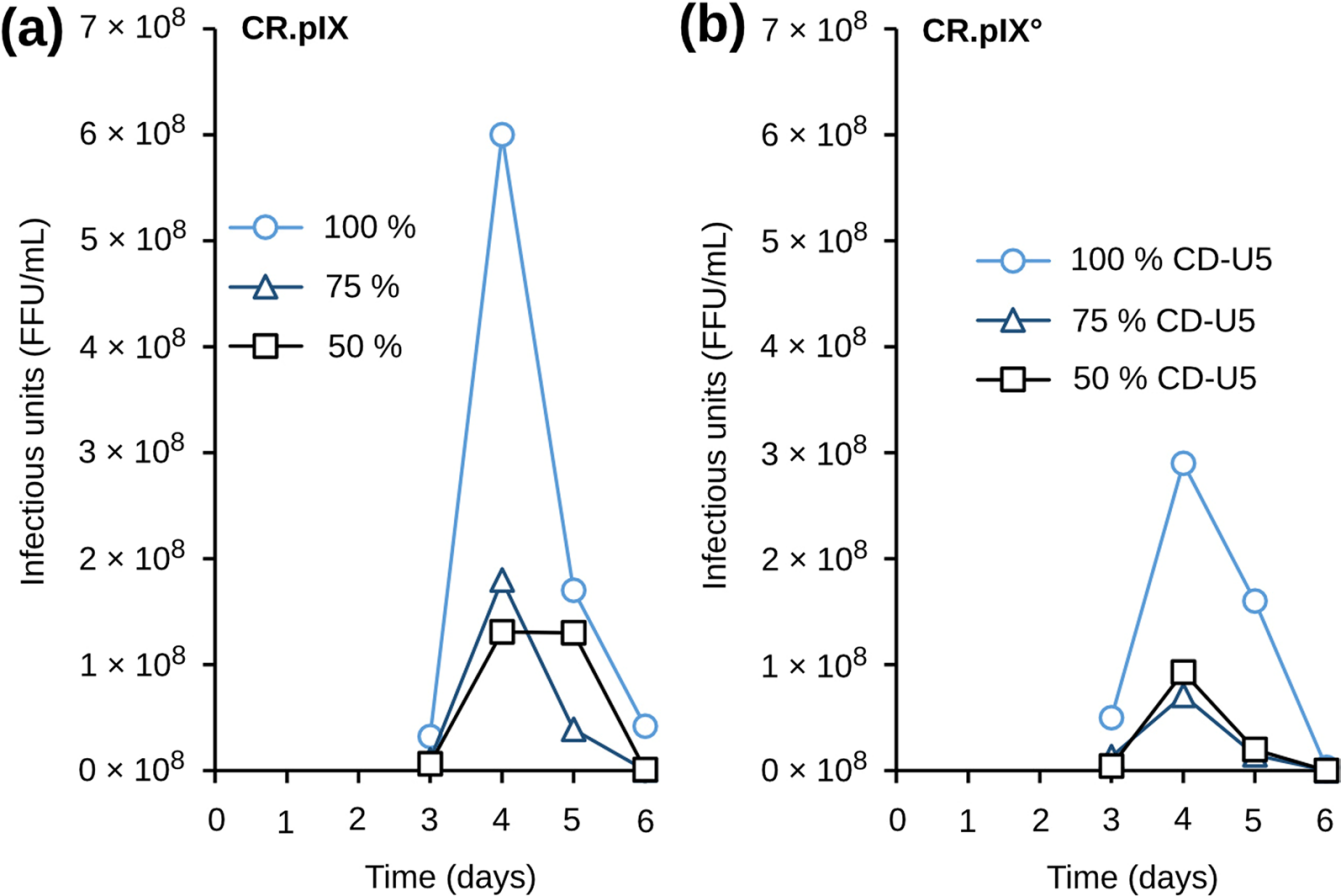
Effect of CD-U5 medium dilution with DMEM on rabies virus productivity. CR.pIX (**a**) and CR.pIX° (**b**) were infected with PV rabies virus strain at an MOI of 0.001. DMEM was added to 50% or 25% respectively (leaving 50% or 75% CD-U5; squares and triangles, respectively). Undiluted CD-U5 suspension culture medium (100%) is shown in light blue (circles)

Figure 5 indicates that both cell lines (CR.pIX and CR.pIX° cells) showed a progressive decrease of specific virus productivity as the percentage of DMEM in the culture medium increased. In 75% DMEM + 25% CD-U5 medium, the specific productivity dropped by 75% compared to the non diluted medium. Hence supplementation of CD-U5 with DMEM medium is not recommended.

### Bioreactor culture in batch culture mode

Most vaccines eventually need to be produced at industrial scales. Experiments were therefore carried out in a 7-l stirred bioreactor to confirm the scalability of the developed process. CR.pIX cells were grown in CD-U5 medium in batch mode and the culture was initiated at 1.5 × 10^6^ viable cells/ml in a working volume of 2.5 l. The glucose level was controlled daily and kept at 3 g/l.

**Fig. 6:**
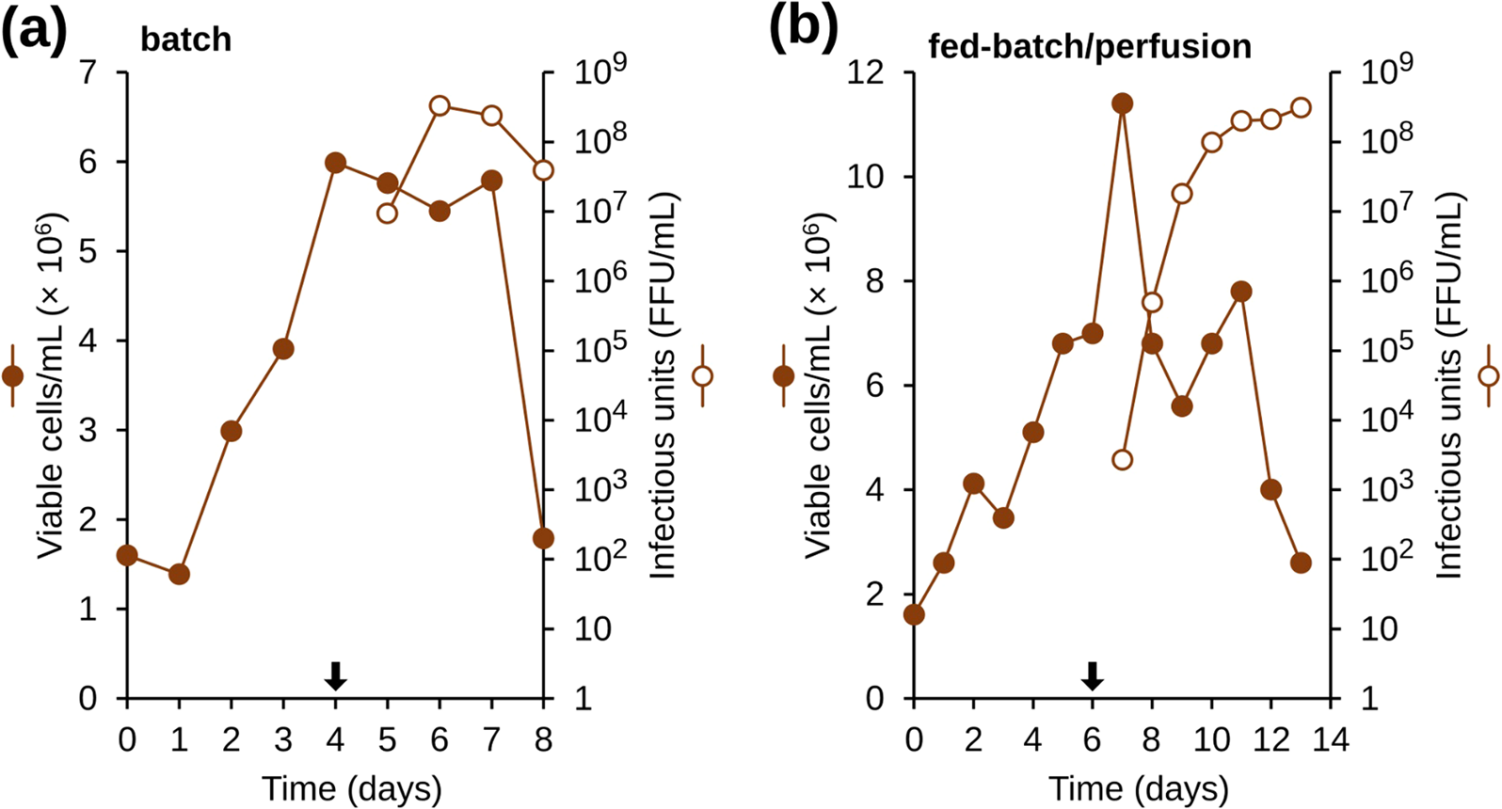
Kinetics of rabies virus production in CR.pIX cells in 7-L bioreactor in (**A**) batch culture, (**B**) fed-batch/perfusion culture. Cells were infected at an MOI of 0.001; the arrows indicate time of infection

As shown in Fig. 6A, viable cell density was 4 × 10^6^ cells/ml at day 3. Temperature was decreased to 34 °C and the cells were infected with an MOI of 0.001 without proceeding to a medium exchange. The cell profile was similar to what was obtained previously with further proliferation until 3 to 4 days post infection, and a decrease of the cell density thereafter. Concurrently, a progressive increase of virus titer was observed. The highest virus titer of 3 × 10^8^ FFU/ml was obtained at day 3 post infection. The virus titer decreased 14-fold at the end of the culture interval and cell viability was around 23%. The viral harvest collected at the end of this experiment was clarified through a 8 µm filter then inactivated by β-propiolactone, as described above. The potency of the experimental vaccine was determined without addition of adjuvant in mice according to the NIH test and was 3.5 IU/ml.

### Bioreactor culture in perfusion mode

CR.pIX cells were grown in 7-l bioreactor with a working volume of 2 l under the operating conditions described in Material & Methods. Daily feeding with 20% of fresh medium was initiated at day 1 post inoculation and was maintained until infection at day 6. Cell density level increased throughout the fed-batch phase and reached 7 × 10^6^cells/ml at the time of infection (Fig. 6B). The average specific growth rate was around 0.014 h^−1^ during this phase. Cells were infected at day 6 with an MOI of 0.001 without medium exchange. Rabies virus was allowed to replicate under perfusion at a constant rate of 0.5 working reactor volumes/day. Single harvests were daily collected.

The kinetics of infectious titers are shown in Fig. 6B. Virus titer increased continuously through the culture and reached a plateau at the end of the culture. The peak virus titer was obtained at day 7 post infection and was equal to 3.1 × 10^8^ FFU/ml. Eight virus harvests of increasing titers were collected with the batch obtained at the end of the culture exhibiting the highest virus titer. Harvests collected from day 4 post infection until the end of the culture interval were clarified, inactivated and then pooled. The potency of the vaccine was assessed in mice and was equal to 7 IU/ml.

## Discussion

Rabies disease is incurable and cause for the highest human death toll among zoonotic infections [24, 25]. This is especially disturbing as the disease can be prevented with appropriate pre- and post-exposure prophylaxis [26–28].

Worldwide, rabid dogs are the major source of human infection [1]. The global strategic plan to end human deaths from dog-mediated rabies by 2030 was launched in 2015. Four organizations, the World Health Organization (WHO), the World Organization for Animal Health (OIE), the Food and Agriculture Organization of the United Nations (FAO) and the Global Alliance for Rabies Control (GARC), have joined forces as *United Against Rabies* to achieve this goal. Expanded dog vaccination in endemic countries is among the major measures aimed at elimination of dog-mediated human rabies [26].

Livestock can also be highly affected by canine rabies in endemic areas. For example, Turkey reports an incidence rate of 0.10 to 3.87 cases/100 000 cattle [29]. Substantially higher incidence has been reported in Tanzania with 12–25 cases/100 000 cattle annually in rural communities [6]. Annual livestock loss was estimated to accumulate to $12.3 million in Africa and Asia [3].

Dog vaccination coverage should be maintained continuously above the threshold of 70% over years to achieve the interruption of canine rabies transmission to humans. This challenge is beyond the capacity of most developing countries mainly due to the lack of resources [5, 10, 26]. A robust and transportable production process may improve local production of rabies vaccines, which in turn may facilitate access to a vaccine and enhance its availability. Such a scenario may help to increase the vaccination rate of canine populations in endemic and poor countries [5, 30].

In this work we studied the replication of PV rabies virus, initially adapted to BHK-21 cells, in avian CR and CR.pIX cell lines. These cell lines are derived from primary cells of a muscovy duck embryo and were used for the replication of different viruses [17, 19–21, 31]. They are adapted to proliferation in single-cell suspensions in chemically defined medium and amenable to batch cultivation or perfusion processes.

We demonstrate in this study that CR and CR.pIX cells are fully permissive for rabies virus. An infectious titer in the range of 10^7^ to 10^8^ FFU/ml was achieved with an MOI as low as 0.01. Similar results were obtained when the cells were grown in the specifically developed culture medium free of the recombinant growth factor LONG R3IGF, the sole protein component in the medium. This result is important because LONG R3IGF is one major cost factor in chemically defined media. Without this factor, costs of goods and medium complexity are further reduced.

The BHK-derived PV rabies virus strain replicated efficiently in single-cell suspensions of the anatine cell line apparently without dependence on virus adaptation. This property is beneficial in the light of a report where the same virus strain required at least three passages to achieve a high titer in BHK-21 cells that were adapted to proliferation in suspended aggregates in serum-free medium [32]. Although a comparison is difficult, rabies virus isolated from a rabid dog required seven passages in BHK-21 for adaptation to efficient replication [33]. The results obtained here with rabies virus are consistent with previous studies where CR and CR.pIX cells were fully permissive for influenza virus types (A and B) [34], poxviral vectors [18, 19], and a diverse spectrum of avian pathogens [20, 31] without adaptation.

The previous experiences also indicated that modified vaccinia Ankara replicates to higher titers in CR.pIX cells compared to parental CR cells [19]. The CR.pIX cell line was generated by stable transfection of established CR cells with an expression plasmid that encodes pIX [20], a minor structural protein of adenoviruses that appears to be involved in stabilizing adenviral capsids against thermal stress. The small adenoviral protein appears to have additional functions [35] and one model that explains augmented replication of viruses unrelated to adenoviruses suggests that pIX may constitutively elevate Hsp90 into an activated state in the avian cell lines [15]. As Hsp90 is involved in the replication of several viruses, including rabies virus [36], a potential connection between pIX and heat shock responses may also explain the increased yields for rabies virus that were observed in this study.

The Multiplicity of Infection (MOI) is another critical process parameter that often needs to be optimized for new virus production processes. Here, we observed that the MOI only slightly affected peak titers for rabies virus in the avian cell line. Interestingly, although titers were usually around 10^8^ FFU/ml for all MOIs, the highest titer was obtained with an MOI of 0.001. This MOI is 100-fold lower compared to previous data generated within our group with BHK-21 or Vero cells as substrate [37, 38] which further demonstrates the high permissivity and productivity of CR.pIX cells for propagation of rabies virus.

Cell density at the time of infection is another critical process parameter. In this work, CR.pIX infection at 2 × 10^6^ cells/ml resulted in the highest cell specific productivity compared to the other densities (1 × and 4 × 10^6^ cells/ml). At 2 × 10^6^ cells/ml, the specific virus productivity was 2.7 or 1.2-fold higher compared to when the cells were infected at 10^6^ or 4 × 10^6^ cells/ml, respectively. These data confirm that the so-called “cell density effect” also impacts rabies virus production in CR.pIX cells. This effect describes an often observed decrease of the amount of virus produced in a variety of hosts at high cell densities [39–41]. However, high cell density processes using CR.pIX cells were successfully developed for MVA [18, 19]. In these studies, medium feeding and exchange strategies applied prior to or during the virus replication phase were demonstrated to restore a high specific productivity. We could show that perfusion during the virus production phase maintained high infectious titers also of rabies virus until the end of the culture and without the drop observed in batch cultures. As an additional advantage, multiple harvests could be collected (Table 1) and the potency of the experimental vaccine was enhanced twofold. Consequently, the expected total vaccine doses that could be produced using perfusion was increased by 9.6 fold (Table 1). This performance was even higher than our previous data obtained with BHK-21 cells grown in serum containing medium in perfusion mode in a 20-l bioreactor [37].

**Table 1:** Rabies virus production in CR.pIX suspension cells grown in a 7-L bioreactor using different culture modes

Culturemode	Timeofculture(days)	Workingvolume(ml)	Harvestvolume(ml)	Virustiter(FFU/ml)	GPlevel(µg/ml)	Activity(IU/ml)	Expecteddoses
Batch	8	2500	2500	2.3 × 10^7^	13 ± 2	3.5	8750
Fedbatch/perfusion	13	4000*	2000	1.9 × 10^7^	2.8 ± 1	7	84,000
			2000	1.3 × 10^8^	32 ± 12		
			2000	3.2 × 10^8^	69.7 ± 5		
			2000	2.5 × 10^8^	73.4 ± 4		
			4000	3.3 × 10^8^	78.6 ± 10		

^*^Working volume during the virus production phase

## Conclusion

We demonstrate in the present study that the CR.pIX suspension cell line in chemically defined medium is permissive for PV rabies virus. The production processes we report here achieved a high virus titer and competitive vaccine potency. This development is a good starting point for optimization of processes for production of veterinary rabies vaccines that are expected to be affordable in endemic countries and may thus contribute to increased vaccination coverages in the rabies reservoirs.

## Methods

### Cell lines and medium

AGE1.CR, AGE1.CR.pIX, AGE1.CR° and AGE1.CR.pIX° cell lines derived from the primary cells of a Muscovy duck embryo, were provided by ProBioGen (Berlin, Germany) and used in this study. Cells were grown in the chemically defined medium CD-U5 (Becton Dickson Biosciences, USA) supplemented with recombinant insulin-like growth factor (LONG-R3IGF, Kerry, USA) to 10 ng/ml, glutamax (Invitrogen) to a final concentration of 2 mM, and NaHCO_3_ to 2.1 g/L. Dulbecco's Modified Eagle Medium (DMEM) and DMEM/F-12 (DMEM/Nutrient Mixture F-12) were provided by Invitrogen (USA). Pilot experiments were performed with adherent CR and CR.pIX cells cultivated in DMEM/F12 and 5% FCS (Gibco #10,086).

### Virus strain

Pasteur virus strain adapted to replication in BHK-21 cells (PV/BHK-21) was obtained from Institut Pasteur (Paris, France).

### Cell suspension culture

Cultures were carried out in shake flasks with baffles (Corning) at 36.5 °C, 8% CO_2_ and 150 rpm in an incubator shaker (Infors AG, Switzerland). CR and CR.pIX were grown in the chemically defined medium CD-U5 supplemented with 10 ng/ml of LONG-R3IGF whereas CR° and CR.pIX° were cultivated in the same medium without the addition of any growth factor.

### Cell infection

Cells were infected at 34 °C, 4.4% CO_2_ and 150 rpm. pH was adjusted to 7.4 by daily addition of NaHCO_3_ at 88 g/l. Infections at small scale were performed concurrent with cell seeding at day 0 of the kinetic. Infectious units were determined with samples cleared by centrifugation.

### Bioreactor cultures

Cultures were performed in a 7-l bioreactor (Sartorius Biobraun, Germany), containing 2 l as a working volume, equipped with a pitch blade impeller and a spin filter (pore size: 10 µm) fixed on the axis. During the cell proliferation step, the following conditions were maintained: pH 7.2 regulated by CO_2_ sparging or addition of NaHCO_3_ at 88 g/l, dissolved oxygen was regulated at 30% air saturation by a continuous surface aeration, temperature was maintained at 37 °C, and the agitation speed set at 150 rpm.

Batch culture was started at 1.5 × 10^6^ cells/ml. For the fed-batch culture, the feed was started after 1 day of culture by daily adding 1/5 working volume. During the rabies virus production phase, pH was maintained at 7.4, pO_2_ at 30% air-saturation and the temperature at 34 °C. After cell infection, perfusion rate was maintained at 0.5 reactor volume/day. Samples were taken daily to determine cell density, cell viability, virus titer, cell infection and glucose level. Cells were counted using a hemacytometer. Cell viability was estimated via the Trypan blue exclusion method.

### Rabies virus titration and immunofluorescence assay

Virus titer was determined in BHK-21 cells according to a modified rapid fluorescence inhibition test (RFFIT) [42]. Serial dilutions of rabies virus were prepared in 96 well plates, BHK-21 cell suspensions were added to the wells, and the plates were incubated at 37 °C, 5% CO_2_ for 20 to 22 h. Cells were washed and fixed by adding cold 80% acetone. Infected cells were detected by immunofluorescence using a fluoresceine-labelled antibody (Bio-Rad #357–2114). Titers are given in Fluorescent Focus Units (FFU/mL).

For immunodetection the cells in 96 well plates were fixed by complete submersion in -20 °C-cold 80% acetone for 10 min. The plates were allowed to dry at ambient temperature and the cell monolayer was rehydrated with PBS. This supernatant was replaced with 20-fold diluted fluoresceine-labelled antibody (Bio-Rad #357–2114) in 50 µl of PBS, incubated for 60 min, washed twice with PBS and photographed with an Olympus IX50/DP73 fluorescence microscope.

### Enzyme linked immunosorbent assay

Rabies virus glycoprotein content in viral harvests was estimated by an indirect enzyme-linked immunosorbent assay (ELISA) as described previously [43].

### Virus harvest inactivation

Rabies virus harvests were first clarified by filtration through 8 μm filter then inactivated by β-propiolactone [final dilution 1/4000 (v/v)] [44].

### Potency test

The protective activity of inactivated rabies virus harvests was determined according to the NIH test [45]. Swiss mice were intraperitonealy immunized at day 0 and 7 and then intracerebrally challenged using the Challenge Virus Standard strain (CVS strain). An internal reference calibrated to the international reference vaccine was also used. The potency is expressed in International Units/ml (IU/ml).

## Data Availability

All data generated or analyzed during this study are included in this published article. The CR.pIX cell line and chemically defined media have been made available for academic research under an MTA.
